# Suicide in the medical community

**DOI:** 10.1192/j.eurpsy.2022.2184

**Published:** 2022-09-01

**Authors:** A. Gonzaga, G. Medina Ojeda, T. Jiménez Aparicio, M. Queipo De Llano De La Viuda, G. Guerra Valera

**Affiliations:** 1 HOSPITAL CLINICO UNIVERSITARIO DE VALLADOLID, Psiquiatria, VALLADOLID, Spain; 2 Sacyl, Hospital Clínico Universitario Valladolid, Psiquiatría, Valladolid, Spain; 3 Hospital Clínico Universitario, Psiquiatría, Valladolid, Spain; 4 Hospital Clínico Universitario de Valladolid, Psychiatry, Valladolid, Spain

**Keywords:** suicidal ideation, doctors, Suicide, physicians

## Abstract

**Introduction:**

Like in the general population, in the medical community the most common mental disorders reported are depression and anxiety. Suicide risk was increased, especially in medical-related professions.

**Objectives:**

To evaluate male and female psysician suicide risk.

**Methods:**

Review all studies involving suicides, suicide attempts or suicidal ideation in health-care workers published in the last five years.

**Results:**

Suicide decreased over time, especially in Europe. Some specialties might be at higher risk such as psychiatrists, general surgeons and anesthesiologists.

**Conclusions:**

Psysicians are an at-risk profession of suicide, with women particularly at risk.

**Disclosure:**

No significant relationships.

